# Relationship between cup position and obturator externus muscle in total hip arthroplasty

**DOI:** 10.1186/1749-799X-5-44

**Published:** 2010-07-21

**Authors:** Michael Müller, Marc Dewey, Ivonne Springer, Carsten Perka, Stephan Tohtz

**Affiliations:** 1 Charité - University Medicine, Center for Musculoskeletal Surgery, Department of Orthopaedics, Charitéplatz 1, Berlin, D-10117, Germany; 2 Charité - University Medicine; Department of Radiology, Charitéplatz 1, Berlin, D-10117, Germany

## Abstract

**Background:**

It is often challenging to find the causes for postoperative pain syndromes after total hip replacement, since they can be very allotropic. One possible cause is the muscular impingement syndrome. The most commonly known impingement syndrome is the psoas impingement. Another recently described impingement syndrome is the obturator externus muscle impingement. The aim of this study is to analyze pathological conditions of the Obturator externus and to show possible causes.

**Methods:**

40 patients who had undergone a total hip replacement were subjected to clinical and MRI examinations 12 months after the surgery. The Harris Hip Score (HHS) was used to analyze pain and function. Additionally, a satisfaction score and a pain score (VAS) were determined. The MRI allowed for the assessment of the spatial relation between the obturator externus muscle and the acetabulum. Also measured were the acetabular inclination angle as well as the volume and cross-sectional area of the obturator externus muscle.

**Results:**

The patients were assigned to 3 groups in accordance with their MRI results. Group 1 patients (n = 18) showed no contact between the obturator externus and the acetabulum. Group 2 (n = 13) showed contact, and group 3 (n = 9) an additional clear displacement of the muscle in its course. It was not possible to establish a connection between the imaging findings, the HHS, the VAS, and patient satisfaction. What was striking, however, was a significant difference between the median inclination angle in group 1 (40° ± 5.4°) and group 3 (49° ± 4.7°) (p < 0.05), and the corresponding image-morphological pathology. The average inclination angle in group 2 was 43.3° ± 3.8°

**Conclusion:**

Contact between the obturator externus muscle and the caudal acetabula border occurs frequently, but is only rarely accompanied by a painful muscular impingement. The position of the acetabula must be seen as one of the main risk factors for contact between the acetabula border and the obturator. The hip replacement process must provide for sufficient osseous coverage of the caudal acetabula border. Furthermore, the retention of the transverse ligament may serve as protective cover for the incisura acetabuli.

## Background

**Figure 1:**
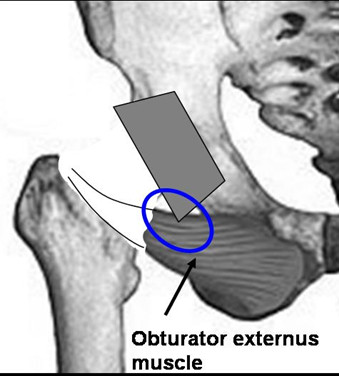
**Graphic illustration of the course of the obturator externus muscle from the obturator membrane to the trochanteric fossa and possible caudal irritation (impingement) at the caudal acetabular border**.

Total hip replacement is one of the most successful orthopedic surgeries, and leads to a high degree of postoperative patient satisfaction. A small percentage of patients, however, experience postoperatively persisting or new symptoms, the causes of which usually present a diagnostic challenge [1,2]. Some of the most common symptom causes are complications such as infections, fractures, dislocations, incorrectly positioned implants, or other underlying pathologies such as degenerative spinal or vascular diseases. Some of the less common causes are muscular impingement syndromes. Very narrow spatial conditions between the muscular structure and the implant lead to chronic irritation and a painful mobility restriction of the hip joint. The psoas impingement is one of the best known impingement syndromes that can occur in connection with an implant [3]. The chronic irritation of the psoas tendon at the anterior acetabula leads to a painful flexion of the hip joint [4]. Caused by the very narrow spatial relations between the whole periarticular hip muscles and the prosthesis further muscle impingement syndromes are conceivable. In a recently reported case, the possibility of an obturator externus muscle impingement was shown [5]. They were able to demonstrate a painful irritation of the obturator externus muscle at the caudal acetabular border in its course from the obturator membrane to the trochanteric fossa (Fig. 1).

We were not familiar with any other studies about an impingement of the obturator externus, and decided to conduct this study to further evaluate the pathology of an obturator externus impingement.

The aim of this study is to research overall spatial relations between the obturator externus muscle and the acetabular components, and to draw conclusions about possibly resulting symptoms. The study will subsequently determine and clarify, if obturator externus impingement does occur and what its possible causes might be.

## Materials and methods

40 patients with total hip replacement (18 men, 22 women) gave written consent and were included in this study. The study protocol was approved by the institutional review board (EA 1/068/06). The average age was 65 years (37-80). The body mass index was calculated at 28 kg/sqm (21-33). Patients were previously checked for common causes which are also responsible for a painful THA. Consequently, patients with verifiable complications such as infections, dislocations, fractures, aseptic loosening, or incorrectly positioned implants were not included. Also not included were patients that suffered from other conditions such as symptomatic degenerative changes of the lumbar spine or vascular diseases. All patients had undergone total hip replacement in our hospital between October 2006 and April 2007. For 34 patients, the reason for the joint replacement was a primary or secondary coxarthritis, and for 6 patients, necrosis of the femoral head. The prosthesis was a cement-free total endoprosthesis that was implanted either through an anterior lateral, or a transgluteal approach. The femur component was either a Zweymüller SL standard shaft (Plus^®^ Orthopedics AG, Rotkreuz, Switzerland) or an Alloclassic shaft (Zimmer^®^, Orthopedics, Winterthur, Switzerland). For the acetabular component, an Allofit^®^ Acetabular press cup system (Zimmer^®^), or a Bicon screw cup system (Plus^®^) were used. The surgeons had aimed to implant the cup in 45° inclination and 15° anteversion and to consider adequate osseous cover. The transverse acetabular ligament was always preserved. All patients underwent general endotracheal anesthesia without any additionally nerval block.

Twelve months after the surgery, the patients underwent a clinical examination and an MRI. The Harris Hip Score was calculated to evaluate pain and function. In addition, a satisfaction score with a scale of 1-6 (1: very satisfied to 6: not satisfied) and a pain score based on the visual analog scale (VAS) (0: no pain to 10: unbearable pain) were obtained.

The MRIs were done on a 1.5 Tesla tomograph (Twin speed, Siemens, Erlangen, Germany,) and by using a quadrature body coil. MR sequences consist of coronal T1-weighted turbo spin-echo (TSE, 667/12 (repetition time msec/echo time msec), 5-mm section thickness, flip angle of 150°, 400 × 400 mm field of view, 512 × 256 matrix), transverse T1-weighted TSE sequence, (667/12, (repetition time msec/echo time msec), 6-mm section thickness, 420 × 275.52 mm field of view, 512 × 168 matrix), and turbo-inversion recovery magnitude (TIRM) coronal T2-weighted fast spin-echo (6040/30/150 (repetition time msec/echo time msec/inversion time msec), 6-mm section thickness, flip angle of 150°, 400 × 400 mm field of view, 512 × 256 matrix). The frequency encoding gradient was always parallel to the long axis of the prosthesis (craniocaudal direction).

The objective of the MRI assessment was the spatial relation between the obturator externus muscle and the acetabular component, obtained by assessing the images layer by layer in all three views. Soft tissue abnormalities such as bursitides, tendinitides, effusions, or other soft tissue changes were also assessed. Also measured were the acetabular inclination angle and the volume of the muscular cross-sectional area of the obturator externus muscle.

**Figure 2:**
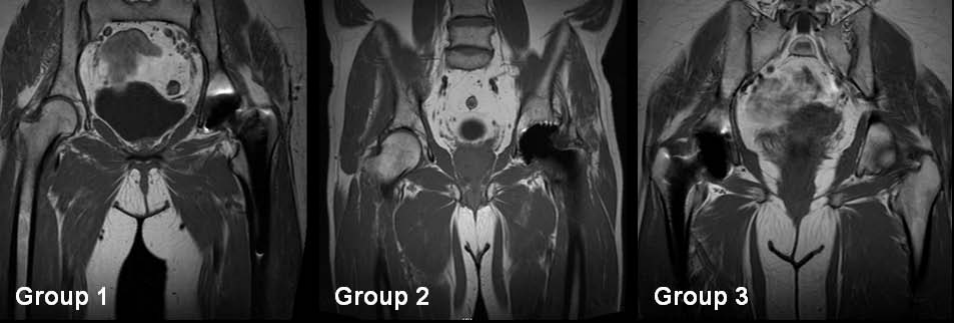
**Example illustration of the spatial relation as shown in the MRI images between the obturator externus muscle and the acetabular component**. Group 1 shows no contact between the obturator externus muscle and the acetabulum, group 2 shows slight contact, and group 3 shows a displacement of the obturator externus muscle in its course.

According to the findings in the MRI the patients were assigned to 3 groups. Group 1 consisted of patients that did not show any contact between the obturator externus and the actetabular component, group 2 consisted of patients that showed visible contact between the caudal rim of the cup and the muscle, and group 3 consisted of patients with contact and an additional clear displacement of the course of the obturator externus muscle through the cup (Fig. 2).

The inclination of the cup was calculated from images on the MR workstation using the anterior pelvic plane as reference and the radiographic inclination as defined by Murray et al. [6]. The determination of the anterior pelvic plane was by means of the MRI scan, where the coronal plane was adjusted in the orientation of the anterior superior iliac spines and the pubic tubercle.

Statistical analysis was performed using SPSS (Version 15, SPSS Inc., Chicago, USA). Pre- and postoperative continuously and normally distributed variables in one group were compared with a Student's t-test. Continuous variables between the groups were compared with the Mann-Whitney U-test (inclination angle, scores). A p-value of less than 0.05 was considered significant.

## Results

**Figure 3:**
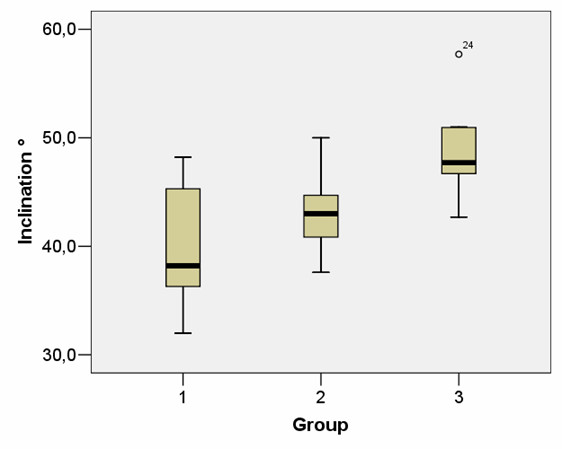
**Illustration of the inclination angle for the respective group**. Correlation between inclination angle and group-specific characteristic of the obturator externus contact. Group 3 showed a median inclination angle of 49 ± 4.7°, group 1, on the other hand, of 40 ± 5.4°.

Forty-five percent (n = 18) of the 40 patients were assigned to group 1 (no contact between the obturator externus and the acetabular cup). 33% (n = 13) showed slight contact (group 2), and 22% (n = 9) additionally showed a displacement of the obturator externus in its course through the acetabular component (group 3). None of the participating patients had any abnormalities or symptoms that could be attributed to an impingement syndrome between the obturator externus muscle and the cup. We were not able to demonstrate a correlation between the imaging findings, mobility range, VAS, and patient satisfaction. The Harris Hip Score was homogeneously distributed throughout the groups. The HHS score's postoperative average value was 90.7 (75 to 99) (Table 1). The average inclination angle for all groups was 43° ± 5.8°. Remarkable was a significant difference between the median inclination angle in groups 1 (40° ± 5.4°) and 3 (49° ± 4.7°) (p < 0.05, Mann-Whitney U-Testing)). The average inclination angle in group 2 was 43.3° ± 3.8° (Fig. 3). The use of a screw cup system or a press system did not impact the likeliness of a muscle-acetabulum contact. The median volume of the obturator externus muscle came to 19  ±  4.2 ccm, and the median cross-sectional area was 824 ± 152 square millimeter. There was no significant difference between the groups in terms of volume and trans-sectional area. Table 1 summarizes the respective data for each group and the overall patient group.

**Table 1:** Demographic data and clinical scores 12 months postoperatively for the respective group.

	Group 1 (no contact)	Group 2 (contact)	Group 3 (contact + displacement)	Total
**Number (n)**	18	13	9	40
**Age (y)**	67 ± 11	68 ± 6.5	56 ± 13	65 ± 11
**BMI (kg/sqm)**	28 ± 2.4	26 ± 3.2	29 ± 4.6	28 ± 3.3
**Inclination angle °**	40 ± 5.4	43.3 ± 3.8	49 ± 4.7	43 ± 5.8
**Volume (ccm)**	19 ± 4.2	20.3 ± 2.5	17 ± 5.6	19 ± 4.2
**Cross-sectional area (square millimeter)**	878 ± 168	861 ± 97	676 ± 88	824.9 ± 152
**HHS preoperatively**	50.6 ± 9	54 ± 13	50.9 ± 7.7	51 ± 10
**HHS postoperatively (12 months)**	92 ± 14	84 ± 16	97 ± 10	90.7 ± 15
**Satisfaction (1-6)**	2 ± 1	2.9 ± 1.9	1.4 ± 0.5	2.2 ± 1.4
**VAS (0-10)**	2.1 ± 1.6	3.6 ± 3.5	1.6 ± 1.1	2.5 ± 2.4

Other soft tissue abnormalities of the obturator externus muscle such as bursitides, effusions, or atrophy were not evident within the study group.

## Discussion

There are very few studies of muscular impingement syndromes after total hip replacement and those that do exist almost always deal with the iliopsoas muscle [3,4,7]. The chronic irritation of the psoas tendon and the resulting iliopectineal bursa at the anterior border of the acetabula lead to a painful restriction of the mobility of the hip [4,8]. Since there are various muscle insertions close to the hip joint, it is principally feasible that other muscles can also be impinged close to the implant. Due to the course of the obturator externus muscle close to the incisura acetabuli and its contact with the transverse acetabular ligament, a pathological contact between the muscle and the border of the acetabula similar to the iliopsoas impingement is feasible. The casuistics described by Müller et al. based on clinical and MRI results and a positive diagnostic infiltration were evidenced as an irritation and inflammation of the obturator externus, and led to the diagnosis of an impingement syndrome [5]. The MRI images were particularly remarkable and indicative, since they showed contact between the acetabular component and the obturator externus muscle, and a displacement of the muscle, accompanied by significant changes of the signals where the muscle makes contact with the acetabula (corresponding to group 3).

The results from this study show, however, that even though in half of the examined patients there is contact between the obturator externus muscle and the lower acetabular rim, the contact does not necessarily lead to any symptoms or pathologies in the examined patients.

These results seem to be in contrast to Müller et al., who could demonstrate a painful contact between the obturator externus muscle and the caudal rim of the cup [5]. Obviously, in most of the cases, a verifiable contact does not result in a painful impingement.

Most of the studies on the psoas impingement describe an accompanying bursitis and tendinitis [7,9,10], which are also responsible for the resulting pain. The iliopectineal bursa is a fixed anatomical component of the psoas muscle and is located immediately adjacent to the acetabular border. It can, therefore, be assumed that the pain felt by patients is most likely the result of the accompanying bursitis and tendinitis. A study by Robinson et al. proved the existence of a bursa of the obturator externus muscle and demonstrated on respective examples that also this bursa could be a reason for painful processes in the hip joint, even though only rarely [11].

In addition, the tendon runs close to the trochanter area and is not located in the immediate vicinity of the acetabulum. Therefore, contact between the acetabulum and the obturator externus muscle is much less likely to cause any symptoms.

Another reason for a more asymptomatic contact of the obturator and the acetabula is the number of respective movement cycles of the hip joint and the respective strength of the muscle contact force. Every day walking and stair climbing make flexion and extension much more common movement processes with verifiable higher muscle contact forces than rotary motions [12,13]. Due to the higher number of movement cycles and more significant force impact, muscle groups involved in flexion and extension such as the iliopsoas are probably much more predisposed for a painful impingement.

Another connection that was discovered in this study is the influence the orientation of the acetabular component has on the development of an impingement. It was possible to show a significant correlation between the inclination angle of the acetabula and the frequency of a muscle-implant contact. The more inclined the acetabular implant, the higher the likelihood of a contact. A large inclination angle should therefore be viewed as a factor of a possible impingement of the obturator externus muscle with the acetabula. Consequently, it is important to make sure that there is sufficient osseous cover of the caudal acetabula border to reduce the risk of an impingement. In this connection, it is absolutely critical to maintain the transverse acetabular ligament during the preparation of the acetabula. The ligament can thus be viewed as a protective anatomic structure between the muscle and the acetabula. The risk of a muscle impingement must also be taken into consideration during a lateralization of the hip center, affected by lateralizing the acetabula, which might become necessary during an offset reconstruction. A lateralized acetabula cup with the possibility of a protruding caudal rim can increase the risk of a pathological contact at the rim [3,8,14]. Therefore, the implantation depth of the acetabula is not absolutely variable.

A correct positioning of the acetabular cup is therefore one of the determining factors in avoiding a muscle impingement, and should always be taken into consideration. Regarding the psoas impingement, it was also possible to show that the positioning of the cup is one of the determining factors for the development of a muscle impingement [3,8].

Overall, unclear postoperative pain after total hip replacement poses a tremendous diagnostic challenge, particularly, when more common causes have been ruled out. The MRI is a new addition to the procedures that are used and is increasingly utilized to evaluate the painful hip replacement due to technological advances and changed prosthesis materials [15,16]. Especially the contrast-rich imaging of the periarticular soft tissue cover and the related, excellent spatial imaging of structural conditions constitute a significant improvement of traditional imaging methods [16]. It allows for the capturing of new pathological connections and insights and an enhancement of the diagnostic spectrum. The study by Pfirrmann et al. shows, for example, that MRI abnormalities can be shown in the periarticular soft tissue cover of many symptomatic hip endoprostheses [15]. Some of the most common findings were bursitides, tendinitis, and muscular and tendinous defects [15]. Going forward, these findings must be included in the evaluation of a painful hip joint and the list of possible causes. These should, as shown by this study, always be viewed in connection with the clinical picture and the patient's symptoms.

The study has some limitations. One limitation of this study is the relative small number of patients which have been enrolled, particularly in the investigation of a rarely existing syndrome. In this connection, a further limiting factor is that rather asymptomatic patients were included than patients with a symptomatic THA. Another disadvantage of the study is that no specific test is available to detect a possible painful obturator externus contact. The outcome was only assessed by means of the Harris Hip Score, a questionnaire of pain and satisfaction. Postoperative patient satisfaction is crucially dependent on the preoperative expectations and pain is a highly subjective measure of outcome [17]. Nevertheless, many authors have reported that pain and function scores are simple, valid, and reproducible measures of patient satisfaction and outcome after THA [18-20].

In conclusion, a contact between the obturator externus muscle and the cup was clearly evident in the MRI in about half of the patients. A correlating effect on pain and function did not emerge. The MRI proven contact only rarely seems to lead to a painful muscular impingement. The reasons can be seen in the frequency of muscle-specific movement cycles and the formation of accompanying painful tendinous and bursa inflammations. The position of the cup, particularly the inclination, and an insufficient osseous cover of the caudal acetabular rim, should be considered a significant risk factor for a contact between the rim of the cup and the obturator muscle.

## Competing interests

The authors declare that they have no competing interests.

## Authors' contributions

MM: conception and design, acquisition of data, analysis and interpretation of data drafting the manuscript. IS: acquisition of data, radiological evaluation. MD: radiological conception and design, radiological evaluation. CP: substantial contributions to conception and design, revising it critically for important intellectual content. ST: analysis and interpretation of data, revising it critically for important intellectual content. All authors have read and approved the final manuscript.
